# Complementary and alternative medicine use and absenteeism among individuals with chronic disease

**DOI:** 10.1186/s12906-016-1195-9

**Published:** 2016-07-27

**Authors:** Jennifer Mongiovi, Zaixing Shi, Heather Greenlee

**Affiliations:** 1 Department of Epidemiology, Mailman School of Public Health, Columbia University, 722 W. 168th Street, Room 703, New York, NY 10032 USA; 2 Herbert Irving Comprehensive Cancer Center, Columbia University Medical Center, New York, NY USA

**Keywords:** Dietary supplements, Mind-body practices, Complementary and alternative medicine, Employee health, Absenteeism, Chronic disease

## Abstract

**Background:**

It is estimated that over half of the adult U.S. population currently has one or more chronic conditions, resulting in up to an estimated $1,600 in productivity loss annually for each employee with chronic disease. Previous studies have suggested that integrating alternative or complementary health approaches with conventional medicine may be beneficial for managing the symptoms, lifestyle changes, treatment, physical and psychosocial consequences that result from chronic illness.

**Methods:**

Using the 2012 National Health Interview Survey Data, we examined the associations between self-reported use of various forms of complementary and alternative medicine (CAM) therapies (dietary supplements, mind-body practices) and the number of days missed from job or business in the past 12 months due to illness or injury. Multivariable Poisson regression was used to determine the association between CAM use and absence from work among individuals with one or more chronic disease (*n* = 10,196).

**Results:**

Over half (54 %) of the study population reported having one chronic disease, while 19 % had three or more conditions. The three most common chronic diseases were high cholesterol (48 %), arthritis (35 %) and hypertension (31 %). More participants used dietary supplements (72 %) while fewer individuals reported using mind-body practices (17 %) in the past twelve months. Over half of individuals reported missing any number of days from job or business due to illness or injury (53 %). Of those who had missed any days from work, 42 % missed one or two days, 36 % missed three to five days, and 23 % missed six days or more. The rate of missing days from job or business due to injury or illness increased among those who reported use of mind-body practices (Incidence Rate Ratio (IRR) = 1.55, 95 % CI: 1.09, 2.21). There was no association between use of dietary supplements and absenteeism (IRR = 1.13, 95 % CI: 0.85, 1.51).

**Conclusions:**

In a population of individuals with chronic disease, individuals who reported use of mind-body practices had higher rate of absenteeism due to injury or illness. Future studies should examine the effects CAM on symptoms associated with chronic disease and whether managing these symptoms can reduce absence from work, school, and other responsibilities.

## Background

It is currently estimated that over half of the adult U.S. population has one or more of the following chronic diseases: hypertension, coronary heart disease, stroke, diabetes, cancer, arthritis, hepatitis, weak or failing kidneys, current asthma, chronic obstructive pulmonary disease [1]. The rate of chronic disease is expected to double the population growth rate by 2020, resulting in 157 million Americans living with at least one chronic illness [2]. By this time, the cost burden of chronic illness is projected to account for over 80 % over of total health spending, including medical costs and job-productivity loss [2, 3].

Chronic conditions have a significant financial impact not only on individuals living with chronic disease but their employers as well. In 2012 it was estimated that lost productivity from absenteeism and presenteeism cost the U.S. economy nearly $1.1 trillion with another $227 billion spent on disease treatment. [4]. Absenteeism is nonattendance from work while presenteeism refers to at-work performance deficits [5]. Reducing absenteeism can save upwards of $1,600 in productivity loss annually for each employee with chronic conditions [6]. U.S. disease treatment costs have the potential to be reduced by over $200 billion [3].

Prior studies have hypothesized that the use of complementary and alternative medicine (CAM) may aid in reducing the stress of chronic disease for employers by improving employee health, therefore, decreasing absenteeism [7, 8]. CAM is often used alongside conventional medicine with the intention of decreasing the severity of both physical and mental symptoms of chronic disease, as well as promoting general well-being [9, 10]. Motives for CAM use include improving health and desire to do something for oneself [11, 12]. CAM healthcare approaches often originate outside of conventional medicine and are practiced together with (complementary/integrative) or instead of (alternative) conventional medicine [13]. These approaches can be provided by a practitioner or self-managed, managing the symptoms, treatment, physical and psychosocial consequences and lifestyle changes that arise from living with chronic disease [14]. Self-managed practices can be used to relieve some of the symptoms associated with chronic disease, improve self-efficacy, and provide individuals a sense of control over and engagement in their own health [14–16].

While many studies have examined the effect of CAM on alleviating symptoms, few have examined how these practices may affect the burden of chronic disease on the population. We used the 2012 National Health Interview Survey (NHIS) data to examine the association between using CAM and the number of days missed from a job or business due to illness or injury by individuals with chronic conditions. We hypothesized that the use of CAM is associated with fewer numbers of sick days taken from work due to engagement in more preventive healthcare behaviors, thus having better health status. Analyses examined the association between CAM use and days missed from job or business due to injury or illness as well as possible predictors of missed days among individuals with chronic conditions.

## Methods

### Survey design

The U.S. Center for Disease Control’s National Center for Health Statistics used a continuous sampling and interviewing design to obtain information on basic demographics, general health, and current health topics through the cross-sectional U.S. National Health Interview Survey (NHIS) [17]. Data were collected from the civilian non-institutionalized United States population, oversampling African American and Hispanic populations. Individuals in the armed forces and those living in institutional group quarters, long-term care institutions, correctional facilities, and countries outside the U.S. were not included. From each household sampled, one adult and one child (if there were any children) were interviewed and provided information on the rest of the household. Beginning in 2002, the NHIS included questions on 27 complementary health approaches every 5 years [18]. The conditional sample adult response rate for the 2012 NHIS was 79.7 % [19].

These analyses made use of the 2012 NHIS. NHIS data are available publically and permission was not required for access. All individuals gave verbal consent prior to participating in this study as indicated in the Field Representative Manual [17]. As this study made use of a de-identified publically available dataset, the study was exempt from IRB oversight by the Columbia University Medical Center IRB.

### Population

A total of 108,131 individuals participated in the 2012 NHIS and 34,525 participants provided data on CAM use. Participants under age 18 and/or were eligible to receive full retirement benefits in 2012 (age 66 and older) were excluded from analysis (*n* = 40,050) [20]. Of the 20 conditions defined as chronic disease by the Multiple Chronic Conditions working group within the Health and Human Services Office of the Assistant Secretary of Health, the 2012 NHIS collected responses on 13 of these conditions [21]. Individuals were included if they answered yes to any of the following: ever having either arthritis, cancer, chronic obstructive pulmonary disease, non-gestational diabetes, hepatitis, high cholesterol, or being informed in the past twelve months of having hypertension, hepatitis, coronary heart disease, asthma, chronic kidney disease, depression, or substance abuse. Participants who did not have one or more chronic disease at the time of the interview (*n* = 43,448), had not worked for pay in the past week (*n* = 10,500), or had reported missing 364 or more days from work in the past twelve months (*n* = 64) were excluded [22]. Working for pay in the past week was the best indicator of employment in the past year opposed to ever having a job. Those who did not or refused to answer any of the survey questions regarding CAM use were excluded from analysis (*n* = 3,873). After removing observations with missing values for the number days missed from job or business in the past twelve months and CAM use the final population size was 10,196.

### Assessment of CAM use

CAM practices considered for inclusion were determined by the National Center for Complementary and Integrative Health’s (NCCIH) definition of “complementary health approaches” for mind and body practices and natural products. Natural products were included in our definition of CAM as these therapies are the most commonly used [9]. CAM practices included acupuncture, massage, meditation, movement therapies (does not include general exercise), relaxation techniques, mind-body practices, vitamins (excluding multi-vitamins), minerals, and herbs [13]. Participants reported not using any of the NCCIH defined practices were considered non-users. CAM types of interest were categorized as either dietary supplements or mind-body practices. Mind-body practices included biofeedback, mantra meditation/mindfulness meditation/spiritual meditation/guided imagery/progressive relaxation, and yoga/tai-chi/qi-gong. Dietary supplements included use of non-multivitamins, non-vitamin supplements, minerals, and herbs.

### Outcome of interest

The primary outcome was the self-reported number of days missed from job or business in the past twelve months due to illness or injury (0–364 days), excluding maternity leave. Participants who had self-reported working in the past week were asked during the past twelve months, “about how many days were missed from a job or business due to injury or illness,” with a possible range of 0–366 days.

### Other variables

Demographic variables included race, age, gender, household income, and highest level of education achieved. A priori hypothesized confounders included BMI (underweight: <18.5, normal: 18.5-24.9, overweight: 25.0-29.9, obese: 30.0+ kg/m^2^), marital status, smoking status, level of alcohol consumption, number of chronic conditions, surgery in the past twelve months, self-perceived general health, health insurance status, number of employees at job or business, class of worker, and reasons for using the self-reported top three CAM therapies. Type of employment was categorized as working for a privately owned company, any type of government (local, state, and federal government), or for one’s self. Possible reasons for CAM use included for general wellness or general disease prevention, to improve energy, to improve immune function, and to improve memory or concentration.

### Analysis

Univariable analysis was performed to determine the raw frequency, weighted percentage, and significance of the association between each variable and either days missed from job/business or CAM use. Using the chi-square test, variables associated with both the main exposure and outcome at α = 0.10 were treated as potential confounders. This α level allowed more variables to be considered for modeling since the effects of these variables on days missed from job or business are not well known. Multivariable Poisson regression was used to evaluate the association between CAM use and days missed from job/business due to illness or injury. The minimally adjusted model included race, age, gender, income, and education as covariates. Potential confounders identified in univariable analyses were included in the fully adjusted models if they modified the beta coefficient of any CAM use in minimally adjusted model by 10 % or greater. Age, BMI, and number of chronic conditions were kept as continuous variables for regression analysis (categorized in Tables 1, 2 and 3). Eigenvalues and variance inflation factors were examined to determine any collinear variables; none of the significant variables were collinear. To make results generalizable to the U.S. population and to adjust for clustering, stratification, and oversampling of specific population subgroups, weighted Poisson regression was performed using final person-level weight which included design, ratio, non-response, and post-stratification adjustments [23]. All analyses were performed using SAS 9.4 (Cary, NC). The SURVEYFREQ commands were used with strata, cluster, and weight to determine weighted percentages. The final model was constructed using PROC GENMOD for weighted regression using Poisson distribution.

**Table 1:** Population characteristics by days missed from job/business due to illness or injury

	Total	Days missed from job or business				*p*-value^a^
		0 days	1 to 2 days	3 to 5 days	6+ days	
	*n* (weighted %)	*n* (weighted %)	*n* (weighted %)	*n* (weighted %)	*n* (weighted %)	
Days missed						
0 days	4781 (46.9)					
1 to 2 days	2199 (22.1)					
3 to 5 days	1898 (19.2)					
6+ days	1296 (11.8)					
Chronic condition^b^						
High Cholesterol	4781 (47.7)	2328 (49.2)	1033 (48.6)	823 (43.4)	597 (47.0)	0.03
Arthritis	3519 (35.2)	1485 (32.5)	767 (34.1)	616 (32.3)	651 (52.7)	<0.0001
Hypertension	3191 (31.2)	1526 (31.4)	645 (28.1)	545 (29.8)	475 (38.3)	0.47
Cancer	1167 (11.9)	560 (12.4)	245 (11.7)	200 (10.5)	162 (12.3)	0.35
Depression	1716 (16.8)	562 (11.2)	366 (17.7)	351 (17.4)	437 (36.3)	<0.0001
Diabetes	1203 (11.2)	593 (12.3)	222 (9.7)	187 (8.3)	201 (14.1)	0.10
Asthma	824 (7.9)	277 (5.8)	171 (7.5)	177 (9.1)	199 (15.1)	<0.0001
Hepatitis	489 (4.2)	255 (4.6)	98 (3.9)	91 (4.3)	45 (3.4)	0.24
Coronary heart disease	253 (2.7)	130 (2.9)	46 (2.4)	26 (1.3)	51 (4.3)	0.55
COPD	192 (1.7)	80 (1.7)	45 (1.9)	38 (1.5)	29 (2.1)	0.93
Substance Abuse	145 (1.6)	69 (1.8)	25 (1.3)	22 (1.2)	29 (1.9)	0.17
Kidney Disease	141 (1.3)	53(1.0)	17 (0.7)	26 (1.6)	45 (3.2)	0.02
Race/Ethnicity						
White	7022 (76.5)	3223 (75.7)	1615 (79.3)	1220 (77.0)	964 (74.3)	ref
African American	1140 (8.9)	532 (9.0)	207 (7.3)	183 (8.0)	218 (12.1)	0.87
Hispanic	1310 (9.8)	652 (10.1)	235 (8.5)	230 (10.3)	193 (10.0)	0.85
Asian/Other	702 (4.8)	374 (5.2)	142 (4.8)	106 (4.7)	80 (3.6)	0.30
Age^c^						<0.0001
18-24	955 (9.9)	428 (9.2)	220 (10.6)	162 (11.0)	145 (10.2)	
25-34	1462 (14.0)	580 (11.7)	331 (15.9)	319 (16.7)	232 (15.5)	
35-44	1998 (19.2)	839 (17.6)	472 (20.2)	359 (20.5)	328 (21.3)	
45-54	2693 (26.7)	1294 (12.7)	569 (26.4)	441 (25.7)	389 (26.4)	
55-65	3066 (30.2)	1640 (34.3)	607 (26.9)	458 (26.1)	361 (26.6)	
Gender						
Male	4419 (44.4)	2205 (47.3)	961 (44.9)	689 (40.7)	564 (38.6)	ref
Female	5755 (55.6)	2576 (52.7)	1238 (55.1)	1050 (59.3)	891 (61.4)	<0.0001
Household income						
<$35,000	2000 (18.2)	961 (18.8)	368 (15.2)	349 (18.7)	322 (20.1)	ref
$35,000-$74,999	3255 (33.2)	1444 (31.6)	722 (34.2)	574 (33.4)	515 (36.7)	0.25
$75,000-$99,999	1621 (16.2)	743 (15.5)	383 (18.0)	268 (15.9)	227 (16.1)	0.75
$100,000+	2879 (32.4)	1426 (34.1)	624 (32.5)	482 (32.0)	347 (27.1)	0.57
Education						
Did not complete high school	858 (7.3)	455 (8.2)	142 (5.4)	133 (7.0)	128 (8.0)	ref
High school diploma or GED	2388 (23.3)	1176 (24.4)	478 (21.5)	376 (21.3)	358 (24.4)	0.65
Associate's degree/some college	3538 (34.8)	1610 (34.1)	753 (33.8)	621 (36.7)	544 (36.1)	0.29
Bachelor's degree	2102 (21.6)	962 (21.2)	527 (24.9)	346 (20.6)	267 (19.2)	0.58
Advanced degree	1248 (13.0)	553 (12.1)	282 (14.4)	260 (14.5)	153 (12.3)	0.24
BMI^c^						0.001
Underweight	56 (0.6)	16 (0.4)	7 (0.2)	10 (0.6)	23 (2.0)	
Normal	2471 (28.9)	1235 (29.7)	546 (29.7)	388 (27.6)	302 (25.9)	
Overweight	3571 (41.4)	1769 (43.2)	771 (41.6)	598 (40.5)	433 (35.5)	
Obese	2531 (29.1)	1119 (26.6)	555 (28.6)	443 (31.3)	414 (36.6)	
Marital status						
Single	2831 (28.0)	1266 (26.4)	573 (27.0)	519 (29.9)	473 (32.9)	ref
Married	5731 (56.8)	2810 (59.5)	1301 (59.2)	921 (53.0)	699 (48.1)	0.0001
Divorced/Separated	1390 (13.3)	591 (11.9)	289 (12.4)	267 (15.5)	40 (16.4)	0.16
Widowed	202 (1.9)	99 (2.1)	35 (1.4)	28 (1.6)	1455 (2.6)	0.66
Smoking status						
Never	5688 (55.1)	2716 (54.6)	126 (56.2)	988 (56.8)	748 (52.9)	ref
Former	2546 (26.3)	1210 (27.7)	577 (26.3)	396 (23.6)	363 (25.0)	0.06
Current	1922 (18.6)	846 (17.7)	381 (17.5)	354 (19.6)	341 (22.1)	0.51
Alcohol consumption^d^						
Abstainer	4230 (39.9)	2054 (41.1)	851 (36.7)	739 (40.7)	586 (39.6)	ref
Light Drinker	3487 (36.2)	1539 (34.2)	793 (38.9)	622 (36.4)	533 (38.5)	0.04
Moderate Drinker	1766 (17.8)	856 (18.7)	409 (18.0)	282 (17.0)	219 (15.4)	0.08
Heavy Drinker	613 (6.1)	288 (6.0)	129 (6.5)	89 (5.8)	107 (6.5)	0.55
Chronic conditions^c^						0.0004
1	5542 (53.8)	2759 (56.4)	1231 (55.2)	924 (53.0)	637 (43.4)	
2	2740 (27.5)	1261 (26.9)	606 (28.4)	457 (27.0)	416 (28.9)	
3+	1892 (18.7)	770 (16.7)	362 (16.4)	35 (20.0)	402 (27.7)	
Surgery in past 12 months						<0.0001
No	4370 (90.4)	4370 (90.4)	1930 (87.7)	1493 (84.5)	877 (61.5)	
Yes	410 (9.6)	410 (9.6)	269 (12.3)	246 (15.5)	576 (38.5)	
Paid sick days						<0.0001
No	6247 (62.5)	2528 (54.4)	1512 (68.8)	1228 (71.7)	979 (68.4)	
Yes	3873 (37.5)	2213 (45.6)	683 (31.2)	503 (28.3)	474 (31.6)	
General health						
Excellent	2464 (25.3)	1265 (27.0)	538 (27.7)	399 (23.3)	262 (18.0)	ref
Very good	3585 (35.6)	1707 (36.0)	825 (36.2)	566 (34.6)	487 (34.4)	0.22
Good	3086 (29.9)	1372 (28.7)	641 (28.5)	568 (31.4)	505 (34.1)	0.005
Fair/Poor	1033 (9.2)	432 (8.2)	195 (7.7)	205 (10.7)	201 (13.4)	0.0002
Health insurance						
No	8690 (86.8)	3983 (84.6)	1931 (88.9)	1687 (90.2)	1089 (85.8)	ref
Yes	1484 (13.2)	798 (15.4)	268 (11.1)	211 (9.8)	207 (14.2)	0.005
Number of employees						
0 to 9	2568 (25.6)	1449 (31.1)	511 (24.1)	311 (17.9)	297 (18.8)	ref
10 to 249	2368 (23.8)	1065 (22.8)	517 (24.1)	462 (27.2)	324 (22.6)	0.0001
50 to 249	2309 (22.9)	954 (20.5)	574 (24.7)	427 (25.0)	354 (25.8)	<0.0001
249 to 9999	1233 (12.7)	529 (11.7)	261 (12.5)	235 (13.5)	208 (15.5)	0.000.
1000+	1435 (14.9)	627 (14.0)	304 (14.6)	263 (16.3)	241 (17.2)	<0.0001
Class of worker						
Private company	7294 (72.5)	3471 (73.7)	1629 (74.3)	1230 (71.3)	964 (66.7)	ref
Government	1879 (18.0)	666 (13.4)	421 (18.4)	409 (22.7)	383 (27.0)	0.06
Self-employed	953 (9.6)	615 (12.9)	141 (7.3)	96 (5.9)	101 (6.3)	0.003
Reasons for CAM use^b^						
General wellness	3140 (47.0)	1439 (45.9)	667 (21.5)	6120 (20.4)	424 (12.3	0.75
Improve energy	1610 (24.0)	713 (44.5)	337 (22.0)	326 (20.7)	234 (12.8)	0.15
Improve immune function	1228 (18.0)	566 (44.4)	247 (21.4)	237 (21.4)	178 (12.7)	0.11
Improve athletic/sports performance	803 (12.0)	371 (44.7)	160 (20.2)	146 (20.2)	126 (14.9)	0.55
Improve memory/concentration	758 (12.0)	328 (42.5)	171 (23.7)	147 (21.6)	112 (12.2)	0.06

^a^Chi-square test for strength of association (α = 0.10)

^a^T- test for strength of association for age, BMI (*p* ≤ 0.10)

Significance for *p*-values was set at 0.05

^b^Groups not exclusive

^c^Modeled as continuous variable

^a^T- test for strength of association for chronic conditions (*p* ≤ 0.10)

^d^Based off the 2010 Dietary Guidelines for Americans [29]

**Table 2:** CAM use in past 12 months

	CAM use^b^			
	Dietary supplements		Mind-body practices	
	*n* (weighted %)	*p*-value^a^	*n* (weighted %)	*p*-value ^a^
Chronic condition^b^				
High Cholesterol	3516 (49.7)	0.26	711 (40.9)	<0.0001
Arthritis	2609 (35.1)	0.23	638 (34.2)	0.92
Hypertension	2395 (46.0)	0.06	433 (22.1)	<0.0001
Cancer	852 (11.3)	0.81	248 (12.5)	0.45
Depression	1242 (15.3)	0.65	503 (25.2)	<0.0001
Diabetes	880 (11.8)	0.67	107 (5.8)	<0.0001
Asthma	597 (8.0)	0.97	252 (12.9)	<0.0001
Hepatitis	350 (3.9)	0.67	106 (4.1)	0.85
Coronary heart disease	175 (2.4)	0.26	35 (2.1)	0.4
COPD	134 (1.5)	0.53	32 (2.1)	0.55
Substance Abuse	109 (1.2)	0.5	47 (2.2)	0.04
Kidney Disease	93 (1.1)	0.38	16 (0.6)	0.15
Race/Ethnicity				
White	5058 (76.5)	ref	1393 (80.3)	ref
African American	837 (9.1)	0.74	145 (5.8)	0.004
Hispanic	927 (9.5)	0.96	166 (7.0)	0.0002
Asian/Other	514 (4.9)	0.26	146 (6.9(	0.44
Age^b^		0.003		0.0005
18-24	679 (12.5)		190 (12.4)	
25-34	1002 (13.1)		374 (18.7)	
35-44	1369 (18.0)		371 (18.6)	
45-54	1957 (27.6)		453 (26.8)	
55-65	2345 (28.9)		462 (23.5)	
Gender				
Male	3040 (44.2)	ref	648 (38.6)	ref
Female	4312 (55.8)	<0.0001	1202 (61.4)	<0.0001
Household income				
<$35,000	1405 (13.6)	ref	384 (14.5)	ref
$35,000-$74,999	2333 (31.1)	0.04	523 (25.8)	0.03
$75,000-$99,999	1168 (17.3)	0.88	261 (14.8)	0.14
$100,000+	2145 (38.0)	0.72	640 (45.0)	0.11
Education				
Did not complete high school	552 (7.2)	ref	67 (3.2)	ref
High school diploma or GED	1634 (22.8)	0.21	246 (15.5)	0.01
Associate's degree/some college	2572 (35.4)	0.005	618 (33.2)	<0.0001
Bachelor's degree	1593 (21.5)	0.005	511 (27.5)	<0.0001
Advanced degree	973 (13.2)	<0.0001	402 (20.6)	<0.0001
BMI^c^		0.003		<0.0001
Underweight	1148 (15.4)		225 (10.6)	
Normal	1856 (24.9)		671 (34.9)	
Overweight	2541 (35.2)		640 (36.0)	
Obese	1807 (24.5)		314 (18.5)	
Marital status				
Single	2068 (28.5)	ref	646 (33.6)	ref
Married	4100 (61.8)	0.06	919 (57.0)	<0.0001
Divorced/Separated	1032 (8.7)	0.28	250 (8.3)	0.01
Widowed	138 (1.1)	0.15	34 (1.1)	0.23
Smoking status				
Never	4167 (57.3)	ref	993 (54.1)	ref
Former	1915 (27.1)	0.85	525 (30.5)	0.08
Current	1255 (15.7)	<0.0001	326 (15.4)	0.24
Alcohol consumption^d^				
Abstainer	968 (15.1)	ref	196 (12.9)	ref
Light Drinker	1728 (26.4)	0.85	428 (25.2)	0.53
Moderate Drinker	1267 (20.6)	0.67	332 (21.9)	0.17
Heavy Drinker	2434 (37.9)	0.52	722 (40.0)	0.10
Chronic conditions^c^		0.19		0.06
1	3901 (53.2)		1070 (58.3)	
2	2021 (28.4)		470 (26.3)	
3+	1430 (18.4)		310 (15.4)	
Surgery in past 12 months				
No	6235 (85.3)	ref	1538 (82.5)	ref
Yes	1114 (14.7)	0.67	310 (17.5)	0.11
Paid sick days				
No	2711 (37.7)	ref	722 (39.4)	ref
Yes	4624 (62.3)	0.13	1126 (60.6)	0.75
General health				
Excellent	1778 (26.5)	ref	515 (31.8)	ref
Very good	2617 (35.5)	0.55	709 (36.1)	0.07
Good	2239 (29.5)	0.79	474 (24.8)	0.0001
Fair/Poor	712 (8.5)	0.06	149 (7.2)	ref
Health insurance				
No	1000 (12.5)	ref	281 (13.9)	ref
Yes	6352 (87.5)	0.01	1569 (86.1)	0.76
Number of employees				
0 to 9	1803 (25.4)	ref	511 (29.9)	ref
10 to 249	1705 (23.4)	0.94	398 (23.0)	0.14
50 to 249	1697 (22.7)	0.57	423 (21.6)	0.10
249 to 9999	869 (12.8)	0.69	185 (10.9)	0.04
1000+	1095 (15.7)	0.05	282 (14.6)	0.26
Class of worker				
Private company	5192 (83.3)	ref	1285 (82.1)	ref
Government	284 (4.4)	0.33	71 (3.4)	0.42
Self-employed	713 (12.3)	0.01	207 (14.5)	0.03
Reasons for CAM use^b^				
General wellness	1980 (27.3)	<0.0001	855 (45.0)	<0.0001
Improve energy	49 (0.5)	<0.0001	38 (1.5)	<0.0001
Improve immune function	184 (2.3)	<0.0001	64 (3.0)	0.004
Improve athletic/sports performance	159 (2.4)	<0.0001	101 (5.7)	<0.0001
Improve memory/concentration	80 (1.4)	<0.0001	33 (2.7)	0.0003

^a^Chi-square test for strength of association (α= 0.10)

^b^Groups not exclusive

^c^Modeled as continuous variable

^d^Based off the 2010 Dietary Guidelines for Americans [29]

**Table 3:** CAM use and days missed from job or business in past 12 months

		Days missed from job or business^b^			
	Total	0 days	1 to 2 days	3 to 5 days	6+ days
	*n* (weighted %)	*n* (weighted %)	*n* (weighted %)	*n* (weighted %)	*n* (weighted %)
CAM use^a^					
Dietary supplements	7336 (72.3)	3425 (46.7)	1551 (21.5)	1413 (19.8)	947 (12.0)
Vitamins^c^	5377 (73.5)	2450 (47.3)	1147 (21.7)	1078 (19.4)	702 (11.6)
Minerals^c^	4337 (57.3)	1978 (48.2)	900 (20.6)	856 (18.5)	603 (12.8)
Herbs^c^	3157 (42.8)	1435 (45.4)	677 (22.5)	626 (19.7)	419 (12.5)
Mind-body practices	1850 (17.3)	722 (41.1)	424 (22.9)	430 (22.1)	274 (13.8)
Yoga^d^	1271 (67.8)	482 (39.6)	320 (25.3)	302 (23.3)	167 (11.9)
Tai-chi^d^	196 (9.3)	69 (34.5)	39 (18.7)	47 (24.0)	41 (22.8)
Qi-gong^d^	84 (3.5)	33 (50.2)	22 (19.9)	16 (17.0)	13 (12.9)
Meditation^d^	877 (47.9)	318 (38.5)	195 (22.0)	209 (22.7)	155 (16.7)
Biofeedback^d^	17 (0.6)	7 (25.1)	2 (4.0)	14 (61.7)	6 (9.2)

^a^Groups not exclusive

^b^Modeled as continuous variable

^c^Percentage of dietary supplement users

^d^Percentage of mind-body practice users

## Results

### Population characteristics

The three most prevalent chronic diseases in this population were high cholesterol (48 %), arthritis (35 %) and hypertension (31 %) (Table 1). Over half (54 %) of the study population reported having one chronic disease, while approximately 19 % reported three or more chronic diseases. Age was limited to adults under 65 years, and the largest group of individuals (27 %) were between the ages of 45 and 54 years. In this sample, 77 % of participants self-identified as white. Over 70 % of the population was above a normal BMI: 41 % were overweight and 29 % were obese. Nearly 10 % of individuals had surgery in the past 12 months. Only 30 % of this working population did not have a minimum education of at least some college. The majority of individuals were employed by a private company (73 %), followed by individuals working for the government (18 %).

### CAM use

In the prior 12 months, most participants reported using dietary supplements (72 %), while only 17 % reported use of mind-body practices (Table 3). The most common dietary supplement was the use of vitamins (74 %), followed by minerals (57 %), and herbs (43 %). Yoga was the most commonly used mind-body practice (68 %) while meditation was the second most common (48 %). Greater percentage of individuals with high cholesterol used dietary supplements (50 %) than mind-body practices (41 %) (Table 2). Similar findings were observed among those with hypertension (46 % vs. 22 %) and diabetes (12 % vs. 6 %). Individuals with depression used mind-body practices more than dietary supplements (25 % vs. 15 %).More individuals reported using mind-body practices for general wellness (45 %) than did users of dietary supplements (27 %).

### Days missed from job or business due to injury or illness

Over half of individuals reported missing any number of days from job or business due to illness or injury (53 %; Table 1). Of those who had missed any days, 42 % missed one or two days, 36 % missed three to five days, and 23 % missed six days or more. The strongest association observed with days missed from work was the highest level of education in both natural supplement and mind-body practice users. Each increase in high level of education achieved decreased the rate of absenteeism (Table 4). Better general health was similarly associated with days missed.

**Table 4:** Association between CAM use and days missed from job or business in past 12 months

	Model 1: Dietary Supplements		Model 2: Mind-body practices	
	IRR (95 % CI)	*p* value^a^	IRR (95 % CI)	*p* value^a^
CAM use				
No	ref	.	ref	.
Yes	1.13 (0.85, 1.51)	0.40	1.55 (1.09, 2.21)	0.01
Race/Ethnicity				
White	ref	.	ref	.
African American	1.08 (0.75, 1.57)	0.67	1.01 (0.65, 1.58)	0.96
Hispanic	0.98 (0.63, 1.52)	0.92	1.34 (0.74, 2.41)	0.33
Asian/Other	1.31 (0.72, 2.41)	0.38	1.12 (0.77, 1.63)	0.55
Age	0.99 (0.99, 1.00)	0.05	1.00 (0.99, 1.00)	0.11
Gender				
Male	ref	.	ref	.
Female	1.06 (0.90, 1.25)	0.48	1.05 (0.88, 1.25)	0.60
Household income				
<$35,000	ref	.	ref	.
$35,000-$74,999	1.23 (0.87, 1.75)	0.24	1.27 (0.90, 1.80)	0.18
$75,000-$99,999	1.17 (0.80, 1.72)	0.41	1.20 (0.83, 1.75)	0.34
$100,000+	1.23 (0.86, 1.77)	0.41	1.26 (0.89, 1.75)	0.34
Education				
Did not complete high school	ref	.	ref	.
High school diploma or GED	0.74 (0.42, 1.30)	0.29	0.73 (0.41, 1.28)	0.27
Associate's degree/some college	0.84 (0.50, 1.45)	0.54	0.89 (0.48, 1.40)	0.48
Bachelor's degree	0.67 (0.39, 1.14)	0.14	0.64 (0.37, 1.09)	0.1
Advanced degree	0.54 (0.32, 0.93)	0.03	0.50 (0.28, 0.88)	0.02
BMI	1.04 (0.70, 1.58)	0.01	1.04 (1.01, 1.07)	0.006
General health				
Excellent	ref	.	ref	.
Very good	1.12 (0.79, 1.55)	0.53	1.12 (0.80, 1.55)	0.50
Good	1.28 (0.93, 1.77)	0.13	1.30 (0.94, 1.79)	0.11
Fair/Poor	1.82 (1.02, 2.61)	0.001	1.80 (1.26, 2.61)	0.001
Reason for CAM use (excludes non-users)				
Improve energy	0.77 (0.40, 2.48)	0.44	0.66 (0.34, 2.38)	0.22

^a^Poisson regression

### Association between CAM use and days missed from job or business

In multivariable Poisson regression adjusting for demographic variables (race, age, gender, income, education) and potential confounders (BMI, general health, and use of CAM to improve energy), the rate of missing days from job or business in the past 12 months among those who used mind-body therapy was significantly greater than those who did not use these practices (IRR = 1.55, 95 % CI: 1.09, 2.21) (Table 4). Similar association was observed for dietary supplements use (IRR = 1.13, 95 % CI: 0.85, 1.51) (Table 4), although the result was not significant.

## Discussion

In a population-based sample of individuals with chronic disease we observed that certain CAM use is associated with increased risk of days missed from a job or business due to illness or injury. Analysis determined that the use of mind-body CAM practices increased the risk missed days. Individuals who had used mind-body practices in the past 12 months reported higher levels of general health than users of dietary supplements. This group also had less risk of absenteeism with increased level of education. Previous studies have reported that meditation in particular has been found to decrease cold and flu illness severity [24, 25]. It has also been reported that those who practice self-managed CAM therapies are less likely to be hospitalized for any reason than those who do not [26]. Decreasing symptom severity and hospitalization may, in turn, decrease absenteeism. Our findings may reflect the health-seeking behaviors among those who are looking to improve their health.

Self-reported general health was associated with the primary outcome (worse health increased risk of absenteeism.), but the validity of this self-reported measure is questionable. Only 9 % of participants claimed their health was less than good (either fair or, poor.), but the overwhelming majority of individuals (70 %) were overweight-obese [27, 28]. This could be attributed to limiting the study population to individuals with one or more chronic condition.

Contrary to previous studies, race/ethnicity was not significantly associated with days missed from job or business. Other studies have reported that African American and Asian ethnicities have been found to have lower vaccination coverage in comparison to those who are white, which may contribute to the number of days missed from work due to illness [29, 30]. Individuals who did not identify as white have also reported more perceived barriers to CAM use [31]. Increasing the highest level of education achieved by an individual decreased the risk of missing any days from work; however, increased annual household income had no effect on this risk. Individuals of lower education and socioeconomic status have previously been found to be more likely to hold a position with low job security (less likely to miss days from work) and higher exposure to workplace hazards (causing injury and illness) [32]. Job security has also been determined to contribute to the higher rate of absenteeism among those in the public compared to private sector [33].

Our study is the first to examine the association between CAM use and absence from work among individuals with one or more chronic disease. Other studies have looked at the effects of specific CAM methods on reducing the burden of chronic disease through the implementation of trials, programs, and guided practices. We chose to look at dietary supplements and mind-body practices since use is common and many of these CAM therapies are self-administered. A larger body of evidence is needed to determine the effects of self-administered CAM practices on wellness. Self-administered CAM practices may be more accessible to individuals with chronic disease regardless of socioeconomic status and other demographic factors. Alternatively, CAM use without the advisement of a practitioner may be harmful due to lack of knowledge on proper technique or dosage. However, there were several limitations to this analysis. The study population was limited to those who were currently employed at the time of the interview. It was not clear whether these individuals were full-time, part-time, temporary, or casual employees. The distribution of days missed from job or business due to illness or injury was skewed left as nearly half of individuals reported not having missed any days from work in the past twelve months (47 %). Knowing employment status and duration of employment may have addressed the issue of non-normality within the data distribution. Although CAM use was limited to vitamins, minerals, herbs, yoga, ta-chi, qigong, meditation, and biofeedback, use of dietary supplements and mind-body practices were the most frequently used CAM methods.

Self-reports of CAM use and days missed from work in the past twelve months were subject to recall bias. This type of information bias may have biased the effects of CAM use on absenteeism towards the null since it is unlikely that classification of either the main exposure or outcome was dependent on the other. Participants with missing data for the main exposure and outcome of interest were excluded from analysis, which may have introduced selection bias if these individuals shared unique, unmeasured characteristics. Although the NHIS is a cross-sectional survey, it is long in length and demands time to complete. Missing information may be due to early termination of the interview. If illness was more common among individuals who were not able to complete the survey, these individuals would have been more likely to report higher numbers of days missed from work. The self-reported days missed from job or business is not dependent on CAM use, so this bias would still be non-differential. Differential misclassification of the outcome was limited by using days missed from job or business as a continuous value instead of collapsing the outcome into categories. Due to the cross-sectional study design, causality cannot be inferred from the observed associations.

### Conclusions

Although nearly all individuals who practiced mind-body therapies reported being in good to excellent health, these individuals had a higher rate of absenteeism compared to non-users. Previous studies have shown that many individuals with chronic illness use CAM with the intent of alleviating the symptoms associated with chronic illness but this body of evidence is limited. Further studies are needed to examine the potential effects of these self-managed CAM therapies on the symptoms associated with chronic disease. Additionally, future studies should explore how managing these symptoms through the integration CAM therapies chronic disease management and employee programs could have a positive effect on absence from work, school, and other responsibilities.

## Abbreviations

CAM, complementary and alternative medicine; IRR, Incidence Rate Ratio; NCCIH, National Center for Complementary and Integrative Health; NHIS, National Health Interview Survey
